# Incompetence of Neutrophils to Invasive Group A *streptococcus* Is Attributed to Induction of Plural Virulence Factors by Dysfunction of a Regulator

**DOI:** 10.1371/journal.pone.0003455

**Published:** 2008-10-21

**Authors:** Manabu Ato, Tadayoshi Ikebe, Hiroki Kawabata, Toshitada Takemori, Haruo Watanabe

**Affiliations:** 1 Department of Immunology, National Institute of Infectious Diseases, Tokyo, Japan; 2 Department of Bacteriology, National Institute of Infectious Diseases, Tokyo, Japan; 3 Laboratory for Immunological Memory, Riken Research Center for Allergy and Immunology, Yokohama-City, Kanagawa, Japan; Yale University School of Medicine, United States of America

## Abstract

Group A *streptococcus* (GAS) causes variety of diseases ranging from common pharyngitis to life-threatening severe invasive diseases, including necrotizing fasciitis and streptococcal toxic shock-like syndrome. The characteristic of invasive GAS infections has been thought to attribute to genetic changes in bacteria, however, no clear evidence has shown due to lack of an intriguingly study using serotype-matched isolates from clinical severe invasive GAS infections. In addition, rare outbreaks of invasive infections and their distinctive pathology in which infectious foci without neutrophil infiltration hypothesized us invasive GAS could evade host defense, especially neutrophil functions. Herein we report that a panel of serotype-matched GAS, which were clinically isolated from severe invasive but not from non-invaive infections, could abrogate functions of human polymorphnuclear neutrophils (PMN) in at least two independent ways; due to inducing necrosis to PMN by enhanced production of a pore-forming toxin streptolysin O (SLO) and due to impairment of PMN migration via digesting interleukin-8, a PMN attracting chemokine, by increased production of a serine protease ScpC. Expression of genes was upregulated by a loss of repressive function with the mutation of *csrS* gene in the all *emm49* severe invasive GAS isolates. The *csrS* mutants from clinical severe invasive GAS isolates exhibited high mortality and disseminated infection with paucity of neutrophils, a characteristic pathology seen in human invasive GAS infection, in a mouse model. However, GAS which lack either SLO or ScpC exhibit much less mortality than the *csrS*-mutated parent invasive GAS isolate to the infected mice. These results suggest that the abilities of GAS to abrogate PMN functions can determine the onset and severity of invasive GAS infection.

## Introduction

*Streptococcus pyogenes* (group A *streptococcus*; GAS) is one of the most common human pathogens. It causes a wide variety of infections ranging from uncomplicated pharyngitis and skin infections to severe and even life-threatening manifestations, such as necrotizing fasciitis (NF) and streptococcal toxic shock-like syndrome (STSS) [1], [2], with high mortality rates ranging from 20% to 60% [3]. Several streptococcal virulence factors, including streptolysin, and M protein, have been reported to be involved in these diseases, by genetic studies or animal-passaged models [1], [2], [4]–[6]. However, which of factors are involved in pathogenesis mediated by clinically isolated severe invasive GAS remains obscure.

The strains of *emm1* genotype, among more than 100 *emm* genes encoding the serotype-determinant M protein, are the predominant cause of severe GAS infections in Japan [7]. Recently, GAS with diverse *emm* genotypes, especially, *emm49*-genotype, have been isolated from patients of severe invasive GAS infections since 2000; however, these genotypes were not isolated before 1999 in Japan [8]. Therefore, *emm49* GAS isolated from invasive infections seems to acquire the novel or altered virulence factors by mutations, genomic additions, or deletions.

Epidemiological and pathological findings, including sporadic incidents of severe invasive GAS infections [9], high frequency of severe invasive infections in immunocompromised host [9], and aggregation of bacteria and a paucity of polymorphnuclear neutrophils (PMN) in foci of invasive GAS infection [10] suggest that host defense factors play an important role in the onset of invasive infections. These findings led us to postulate that invasive GAS infections hampered host innate immune defense, especially on PMN, providing the front-line defense against GAS infection by quick recruitment to infection site and clearance of bacteria following phagocytosis [11], [12]. So far, using animal-passaged GAS mutants, gene-manipulated GAS, many virulence-associated molecules are pointed out to play some roles in the bacterial evasion from phagocytic ingestion by neutrophils [13]. However, restricted availability of clinical isolates with the same serotypes fail to elucidate direct relationship between definitive genetic changes in clinically isolated severe invasive GAS and the lack of PMN at the site of bacterial growth.

In the present study, we aimed to explore the crucial factors in the pathogenesis of severe invasive GAS infections in the context of PMN-GAS relationship, using a panel of *emm49* clinical isolates from patients with or without severe invasive infection, and their gene-manipulated mutants. We now show a direct and previously unrecognized link between functional loss of a factor CsrS of two-component sensor/regulator system (CsrS/CsrR: also known as CovS/CovR) and escape from killing by PMN via inducing necrosis to them and digesting IL-8, a PMN-attracting chemokine. We further determined CsrS mutations in the severe invasive GAS was essential to control the expression of various virulence genes and contributed to the *in vivo* virulence and disease-specific pathophysiology in a mouse model. These data may participate in prediction of GAS potential for future invasive infection as well as risk assessment of patients by measuring PMN function.

## Results

### Group A streptococcus isolates from severe invasive infections is resistant to killing by human PMN

**Figure 1:**
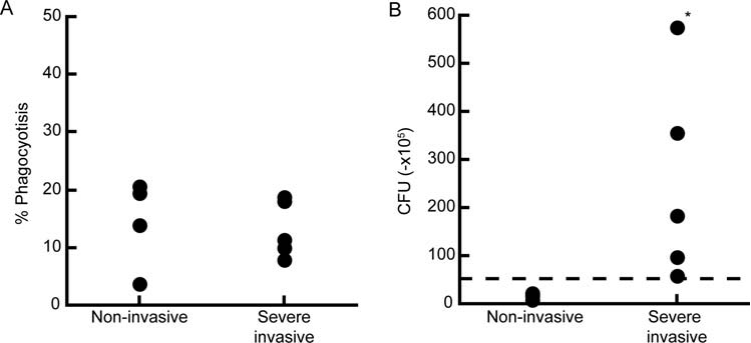
Severe invasive GAS evade killing activity of PMN. (A) Phagocytosis activity of PMN to *emm49* GAS. Four non-invasive strains and 5 severe invasive strains labeled with Alexa-488, followed by incubation with PMN at MOI 10 for 60 min. The proportion of GAS-phagocytosed PMN was then analyzed using flow cytometry. (B) Severe invasive or non-invasive strains were incubated with PMN at MOI 10. After 2 hours, the killing capacity of PMN was estimated by counting the number of bacteria colonies. The dotted line indicates the number of bacteria applied to the culture.

To examine whether *emm49* GAS isolated from severe invasive infection might alter human PMN function, we performed phagocytosis assay *in vitro*. As non-opsonized GAS was resistant to the phagocytosis by PMN [14], we opsonized GAS with human plasma in advance to the assay. As shown in Figure 1A, there was no significant difference between GAS that were isolated from non-invasive and severe invasive infections in phagocytosis by PMN (p = 0.5556). However, as shown in Figure 1B, *in vitro* killing assay revealed that PMN killed non-invasive GAS, resulting in 15–42% of initial number of bacteria, but not invasive GAS (*p* = 0.019). The similar results were obtained when opsonized with either FCS or human serum regardless of complements immobilization (data not shown). These results were common among all PMN donors. These data indicated that clinically isolated severe invasive GAS were phagocytosed, but escaped from killing by human PMN.

### Severe invasive GAS rapidly induce necrosis to human PMN

**Figure 2:**
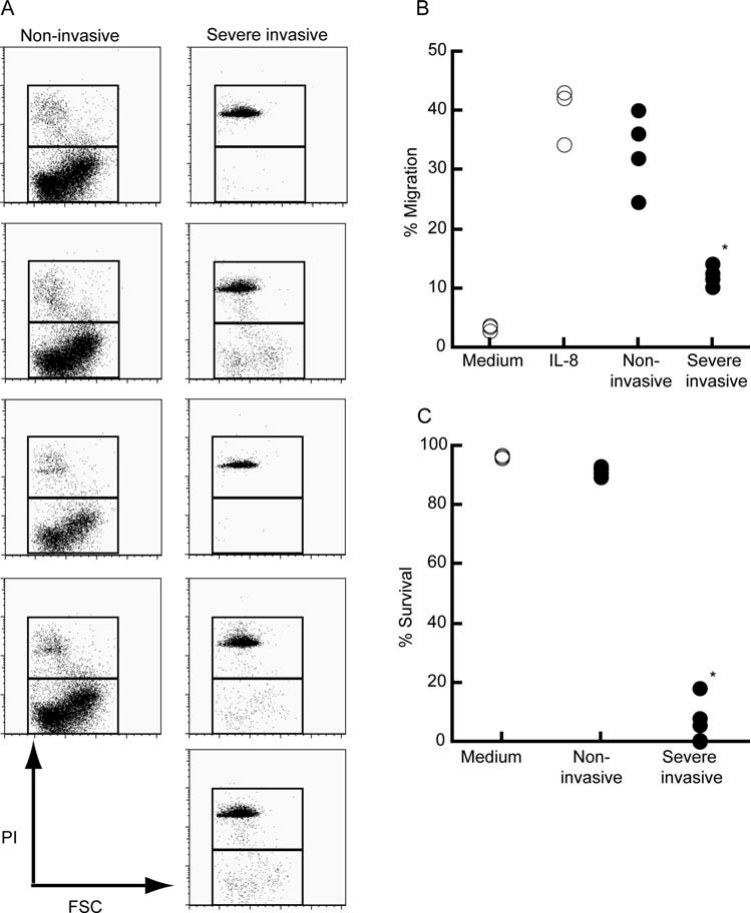
Severe invasive GAS kill human PMN and impair migration ability in response to IL-8. (A) Flow cytometry profiles of migrated PMN in response to 100 nM IL-8 plus GAS. PMN were applied into the upper well (5×10^5^ cells) and those migrated into the lower well in a transwell system in response to IL-8 in the presence of GAS (5×10^6^ CFU) were stained with propidium iodine and were analyzed using flow cytometry. Each panel represents migrated PMN encountered with individual clinically isolated GAS strain. The representative data are shown. (B) The proportion of PMN that migrated into lower wells in response to IL-8, in the presence (closed circles) or absence (open circles) of invasive or non-invasive GAS strains. Total cell numbers consisted of both viable and dead cells were estimated 60 minutes after incubation. (C) The proportion of live PMN that migrated into lower wells in response to IL-8 alone (open circles) or IL-8 in the presence of severe invasive or non-invasive GAS strains (closed circles). Viable cell numbers were analyzed at 60 minutes incubation. *p<0.05 estimated by Mann-Whitney's U test.

In an acute bacterial infection, PMN were quickly recruited at the site of infectious foci according to the gradient of chemoattractants. Therefore, we examined whether GAS clinically isolated from severe invasive infections could affect the migration ability of PMN in response to chemokines. As a model of local infection of the initial phase, we utilized a transwell system and added IL-8 and GAS in culture medium within the lower wells. PMN were applied in the upper wells and subsequently incubated for 90 min. As shown in Figure 2A and 2B, a substantial number of PMN, consisted largely of viable cells, was detected in the lower wells consisted of IL-8 and non-invasive GAS, as a control. Contrarily, number of PMN was significantly low in the presence of severe invasive GAS (*p* = 0.016) compared to that of control culture. Flow cytometry analysis suggested that although PMN was detected in the lower well consisted of IL-8 and severe invasive GAS, but most of them were dead as defined by propidium iodine staining (*p* = 0.016) (Figure 2A and 2C) demonstrating that severe invasive GAS affected survival of PMN and its migration activity in a transwell system.

### PMN were killed by streptolysin O (SLO) from severe invasive GAS

PMN death was induced shortly after encounter with severe invasive GAS, and PMN were not in apoptotic death because of low frequency of cells positive for annexin V (data not shown), which was seen in the case of cytolysin-dependent cell injury [15]. GAS produce two cytolysins that may contribute to pathogenesis. Streptolysin S (SLS) is an oxygen-stable β-hemolysin and Streptolysin O (SLO) is a pore-forming cholesterol-binding toxin [16]. Therefore, to know the mechanism underlying GAS-mediated killing of PMN, we investigated whether SLO produced by invasive GAS affect survival of PMN in an *in vitro* migration assay system.

**Figure 3:**
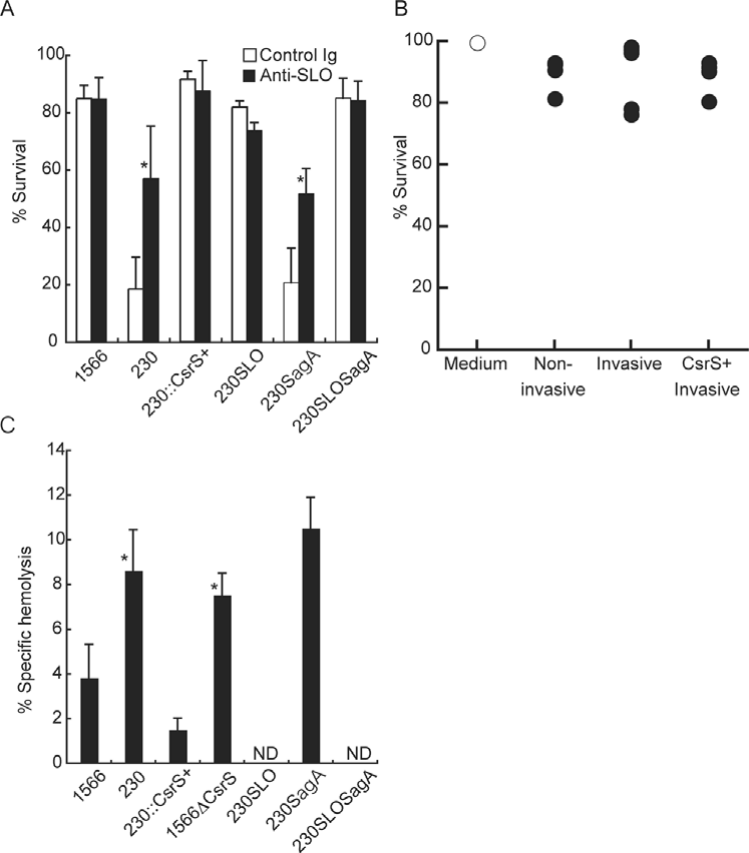
Severe invasive GAS killed PMN by SLO in a contact-dependent manner. (A) The viability of viable PMN that migrated in lower wells of transwell system was estimated as Figure 2. To investigate the role of SLO in PMN survival, lower wells consisted of IL-8 in the presence of either polyclonal rabbit anti-SLO antibodies (25 µg/ml at a final concentration, closed column) or control rabbit IgG (open column), together with either non-invasive GAS (1566), invasive GAS (NIH230), an csrS-transduced NIH230 (NIH230::*csrS*+), an SLO deficient NIH230 mutant (NIH230*slo*), an SLS deficient NIH230 mutant (NIH230*sagA*), or SLO and SLS double mutant (NIH230*slosagA*). Values are mean±SD. *p<0.05 estimated by Student's t-test. (B) No substances secreted from GAS reduced PMN viability. PMN was incubated for 2 hours with supernatants from co-culture of IL-8 and invasive or non-invasive GAS and live cell number was examined as described in Figure 2C. (C). SLO-specific hemolytic activity for sheep erythrocytes in supernatants from overnight culture of invasive or non-invasive GAS as listed in (A). Forty-five minutes after incubation, the absorbance of culture supernatants was measured at 540 nm and the SLO-specific hemolytic activity was calculated as described in methods and presented as the mean±SD. The results represent one of two independent experiments. *p<0.05 significantly higher than non-invasive strain 1566 estimated by ANOVA. ‘ND’ represents less than 0.5% hemolysis.

Figure 3A shows that PMN killing by invasive GAS was blocked by anti-SLO Ab in culture medium within lower wells of a transwell system (p = 0.018 compared with control Ig), at similar extent by adding free cholesterol in the medium (data not shown). Furthermore, an SLO deficient mutant from a STSS isolate NIH230 (NIH230*slo*) lost the killing activity for PMN (Figure 3A), thereby, strongly suggesting that SLO is a key element for PMN killing mediated by invasive GAS. Contrarily, SLS-deficient mutant from NIH230 strain (NIH230*sagA*) killed PMN, as efficiently as did parent strain, indicating that SLS is dispensable for killing of PMN mediated by invasive GAS. SLO and SLS double mutant GAS from NIH230 strain (NIH230*slosagA*) displayed the killing activity indistinguishable from that of NIH230*slo*, confirming the primarily role of SLO for GAS-mediated PMN killing. As shown in Figure 3B, incubation of PMN with supernatant from co-culture of IL-8 and invasive or non-invasive GAS did not affect PMN viability, suggesting that severe invasive GAS causes PMN killing following encounter with bacteria in a contact-dependent manner.

To confirm that SLO activity is increased in invasive GAS strain, we measured SLO hemolytic activity of GAS strains used in this study. As shown in Figure 3C, SLO activity of severe invasive isolate NIH230 is increased as twice as that of non-invasive strain 1566 (p = 0.017).

### Impaired migration of PMN is due to degradation of IL-8 by serine proteinase ScpC

**Figure 4:**
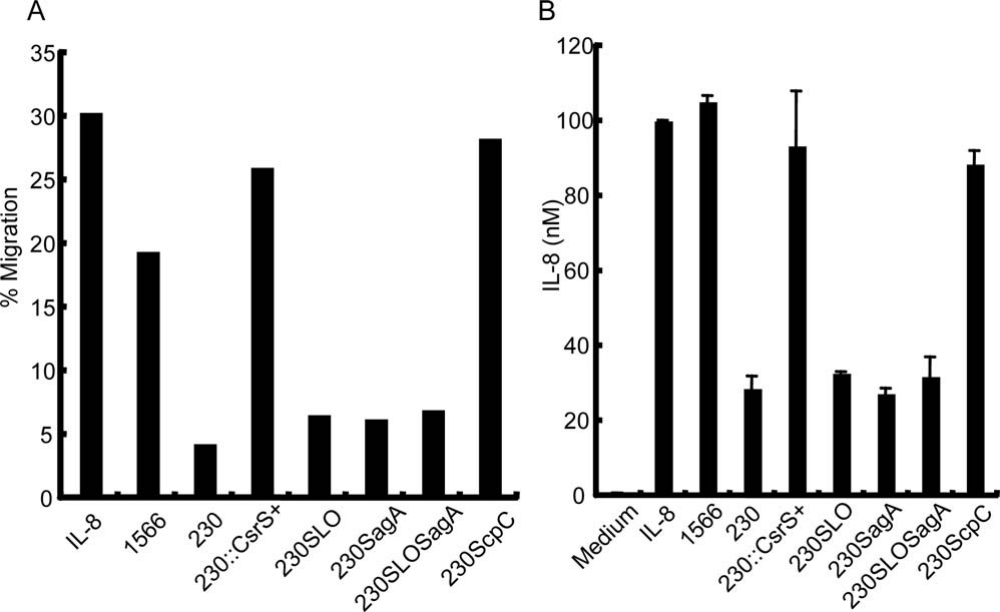
Severe invasive GAS degrade IL-8 by serine protease ScpC, resulting in impaired PMN migration. (A) Migration abilities of PMN in response to IL-8 in the presence of non-invasive and invasive GAS, as listed in Figure 3A plus ScpC deficient NIH230 mutant (NIH230*scpC*). PMNs that migrated into the lower well in a transwell system were estimated by flow cytometry. (B). IL-8 was added into the culture medium (100 nM at a final concentration) and incubated with invasive or non-invasive GAS as listed in (A). Sixty minutes after incubation, the amount of IL-8 in triplicates was measured by sandwich ELISA and presented as the mean±SD. The results represent one of two independent experiments.

Although NIH230*slo* lost the killing activity for PMN, migration of PMN in response to IL-8 in a transwell system was not restored in the presence of this mutant (Figure 4A), thereby, suggesting that severe invasive GAS blocks PMN migration by influence on IL-8 activity. Therefore, we quantified the amount of IL-8 in culture before and after co-culture with clinically isolated GAS or its mutants. Figure 4B shows that the amount of IL-8 was significantly reduced by 60-min co-culture with NIH230, as well as NIH230*slo*, but not with non-invasive GAS 1556. As previous reports suggested that the GAS envelope peptidase ScpC (also known as SpyCEP) degrades the CXC chemokines, such as human IL-8, Groα, murine KC and MIP-2 [17]–[19], we established a NIH230 mutant deficient with ScpC (NIH230*scpC*) and analyzed the property in a PMN migration assay. The results showed that NIH230*scpC* neither digested IL-8, like 1566 strain, (Figure 4B) nor abrogated the migration of PMN in response to IL-8, comparable to 1566 strain (Figure 4A), whereas the mutant killed the migrated PMN as well as the parent strain NIH230 (data not shown). These results demonstrate that clinically isolated invasive GAS impaired PMN recruitment and its survival, as a result of productions of ScpC and SLO, respectively.

### Enhanced expression of the slo and the scpC genes in severe invasive GAS is attributed to mutation of a transcriptional regulator CsrS

**Figure 5:**
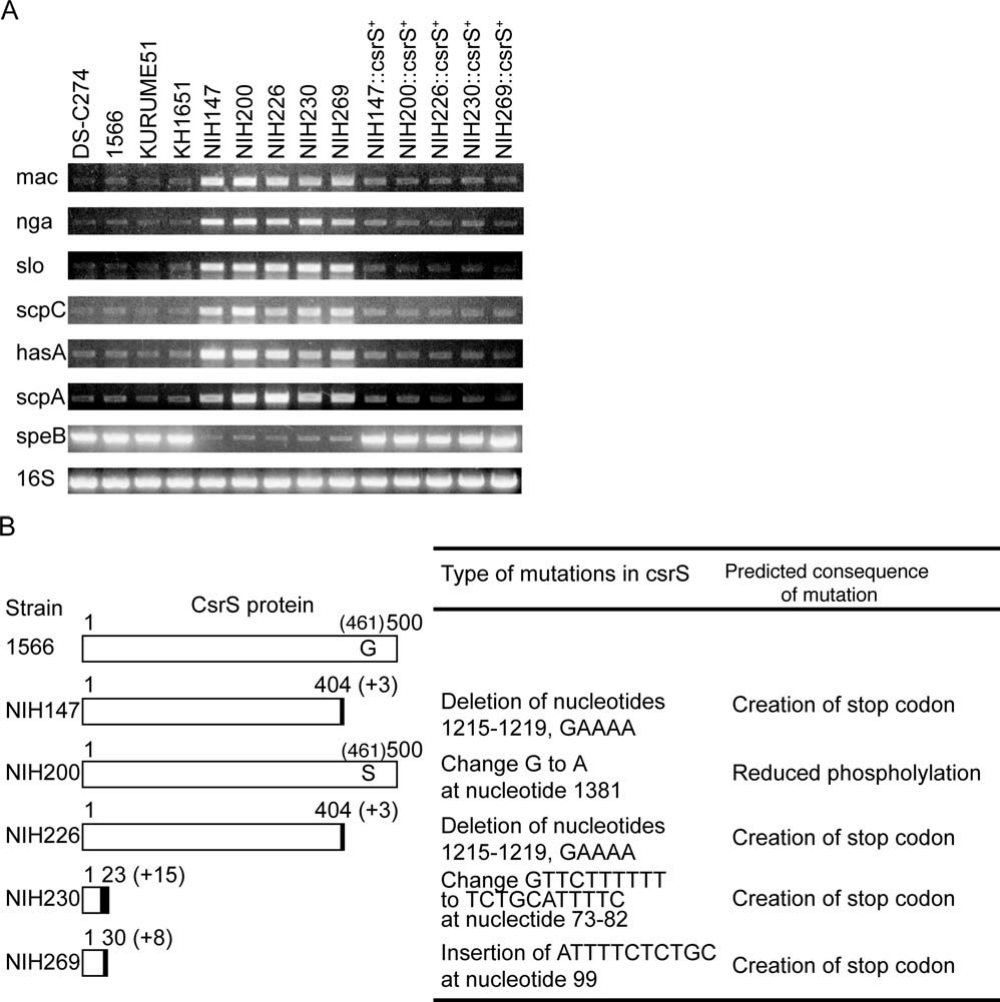
Mutation of the *csrS* gene in the isolates of the patients with severe invasive infections is responsible for increased virulence of GAS. (A) Expression of virulence-associated genes in non-invasive and invasive GAS isolates and mutants transduced with *csrS*, analyzed by RT-PCR. The expression of virulence-associated factors mRNA: IgG degrading protease of GAS, Mac-1-like protein (*mac*), nicotine adenine dinucleotide glycohydrolase (*nga*), *slo*, *scpC*, polysaccharide capsule production (*hasA*), C5a peptidase (*scpA*), and streptococcal pyrogenic endotoxin (*speB*), plus expression of 16S rRNA (*16S*) were shown. (B) The *csrS* mutations in GAS isolates from the patients with severe invasive streptococcal infections. The numbers at the end of the bars indicate the total amino acid residues of CsrS proteins from the start codon in non-invasive GAS (1566) and invasive Gas from the patients (NIH147, NIH200, NIH226, NIH230 and NIH 269). Solid boxes represent the newly created amino acids as a result of frameshift mutations, with length of amino acids ( “+ number” within parentheses). In the NIH200 strain, Ser replaced Gly at position 461 of the CsrS protein. Type of mutations are listed at the end of bars.

Although sequences of the *slo* gene and the *scpC* gene were identical among clinically isolated non-invasive and severe invasive GAS (data not shown), Figure 5A shows that the *slo* and the *scpC* genes were expressed in the severe invasive GAS greater in extent than those in the non-invasive GAS. The expression of the other virulence-associated genes, such as IgG degrading protease of GAS, Mac-1-like protein (*mac*), nicotine adenine dinucleotide glycohydrolase (*nga*), polysaccharide capsule production (*hasA*), and C5a peptidase (*scpA*), was also upregulted in the severe invasive GAS, greater than that detected in the non-invasive GAS (Figure 5A). Contrarily, the levels of streptococcal pyrogenic endotoxin (*speB*), SLS (*sagA*), and mitogenic factor (*speF*) genes were downregulated in the severe invasive GAS, compared to that found in the non-invasive GAS (Figure 5A and data not shown). These results demonstrate the prominent changes in the transcriptional profile of several virulence-associated genes, including the *slo* and the *scpC*, in the all severe invasive GAS.

**Figure 6:**
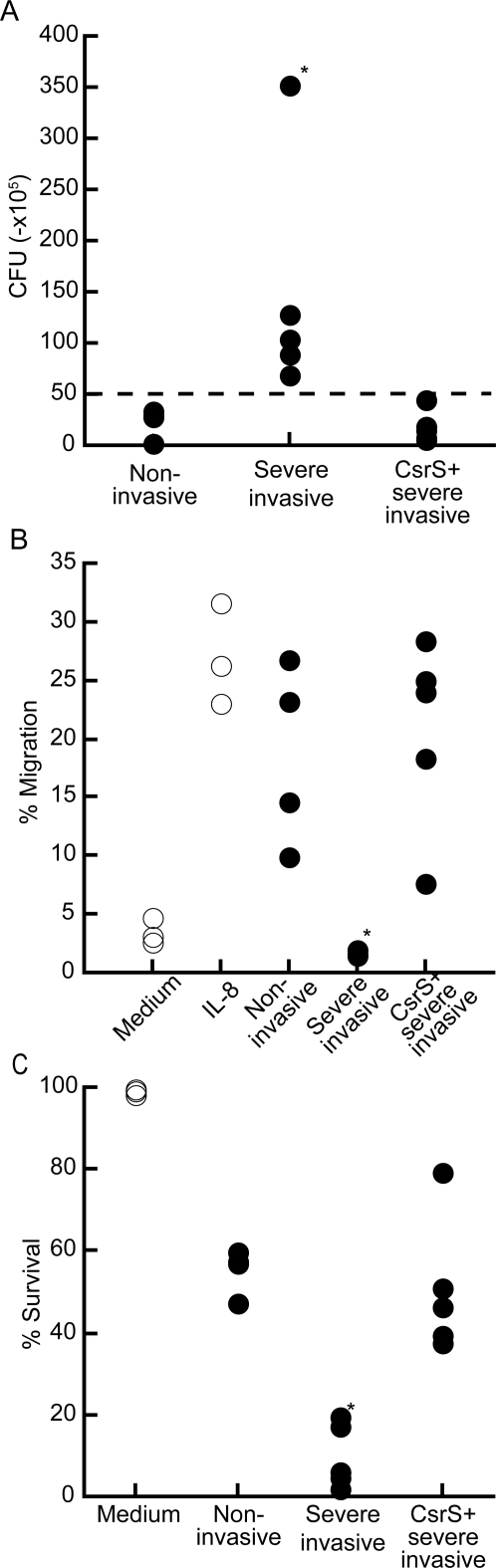
Mutations of CsrS is responsible for increased virulence of GAS to PMN functions. (A) Non-invasive, severe invasive, and invasive strains with overexpression of CsrS strains were incubated with PMN at MOI 10. After 2 hours, the number of live bacteria was counted. The dotted line indicates the number of bacteria applied to the culture. (B)–(C) CsrS transduction into the invasive GAS isolates abrogated the killing activity for PMN as well as the inhibitory effect on PMN migration in a transwell system. (B) The proportion of PMN consisted of both viable and dead cells and (C) The proportion of live PMN that migrated into the lower wells in response to IL-8, in the presence (closed circles) or absence (open circles) of non-invasive GAS, severe invasive GAS isolates or severe invasive GAS isolates transduced with CsrS was analyzed at 60 minutes incubation, as described in Figure. 2B and 2C. *p<0.05 estimated by ANOVA.

Mutation of *csrR* or *csrS* can cause significant alterations in virulence in mouse models of infection,either increasing lethality or the severity of localized soft tissue lesions [5], [6]. GAS isolates from mice with severe invasive disease had mutations in *csrS*, raising the notion that CsrR/S function is important in modulating gene expression during infection. Therefore, we analyzed the linkage between the *csrS* and/or *csrR* genes and the property of invasive GAS infection by sequencing these genes in the *emm49* strains used in this study. The nucleotide sequence of the *csrR* gene was identical in all the isolates, and that of the *csrS* gene was identical among the all non-invasive GAS isolates (data not shown). However, as shown in Figure 5B, the *csrS* genes of all clinically isolated severe invasive GAS had a deletion, a point mutation, or an insertion, thereby, resulting in the creation of translational stop codons (NIH147, NIH226, NIH230, and NIH269) or in a mutation in the presumed kinase domain (NIH200). In order to clarify the role of CsrS regarding expression of the virulence-associated genes and resistance to PMN killing, we introduced the intact *csrS* gene of the 1566 strain into the severe invasive GAS (see Figure 5A). The *csrS*-introduced severe invasive GAS reduced the expression levels of the *slo* and the *scpC* genes, comparable to those detected in the non-invasive GAS. In contrast, the expression of *speB* was upregulated to the level observed in the non-invasive GAS (Figure 5A). In parallel with the expression profile of *slo* and *scpC* in the severe invasive GAS, introduction of the intact *csrS* gene into the severe invasive GAS restored the susceptibility to the killing by PMN (*p* = 0.015 compared with severe invasive isolates +CsrS) (Figure 6A), abrogated the inhibition of PMN migration by degradation of IL-8 (*p* = 0.002 compared with invasive isolates +CsrS) (Figure 4A, 4B, and 6B), and diminished the killing activity for PMN by necrosis (*p* = 0.00016 compared with invasive isolates +CsrS) (Figure 3A, 3C, and 6C), These results strongly suggest that mutations in the *csrS* gene correspond to the immunocompromized activity in the severe invasive isolates, associated with inhibition of PMN recruitment and survival.

### *csrS* mutation is important in the pathogenesis of invasive infections in a mouse model

In order to elucidate the role of *csrS*, in infections *in vivo*, we compared the virulence of GAS isolates using a mouse model which infected GAS intraperitoneally. The non-invasive 1566 strain displayed the LD_50_ value approximately 100-fold higher than that of the severe invasive NIH230 strain (Table 1), whereas a *csrS* deletion (1566Δ*csrS*) caused an increase in the LD_50_ value comparable to that of the NIH230 strain. Consistently, an introduction of the intact *csrS* gene into the NIH230 strain (NIH230::*csrS^+^*) reduced the LD_50_ value to the level observed in the non-invasive strain. These results indicate that *csrS* is an important virulence factor in the mouse model of lethal infections.

**Table 1:** LD_50_ values of each strain.

Strain	LD_50_ value
1566	1.03×10^8^
NIH230	1.11×10^6^
NIH230::*csrS* ^+^	1.52×10^8^
1566Δ*csrS*	8.60×10^5^
NIH230*slo*	3.33×10^7^
NIH230*scpC*	1.04×10^7^

**Figure 7:**
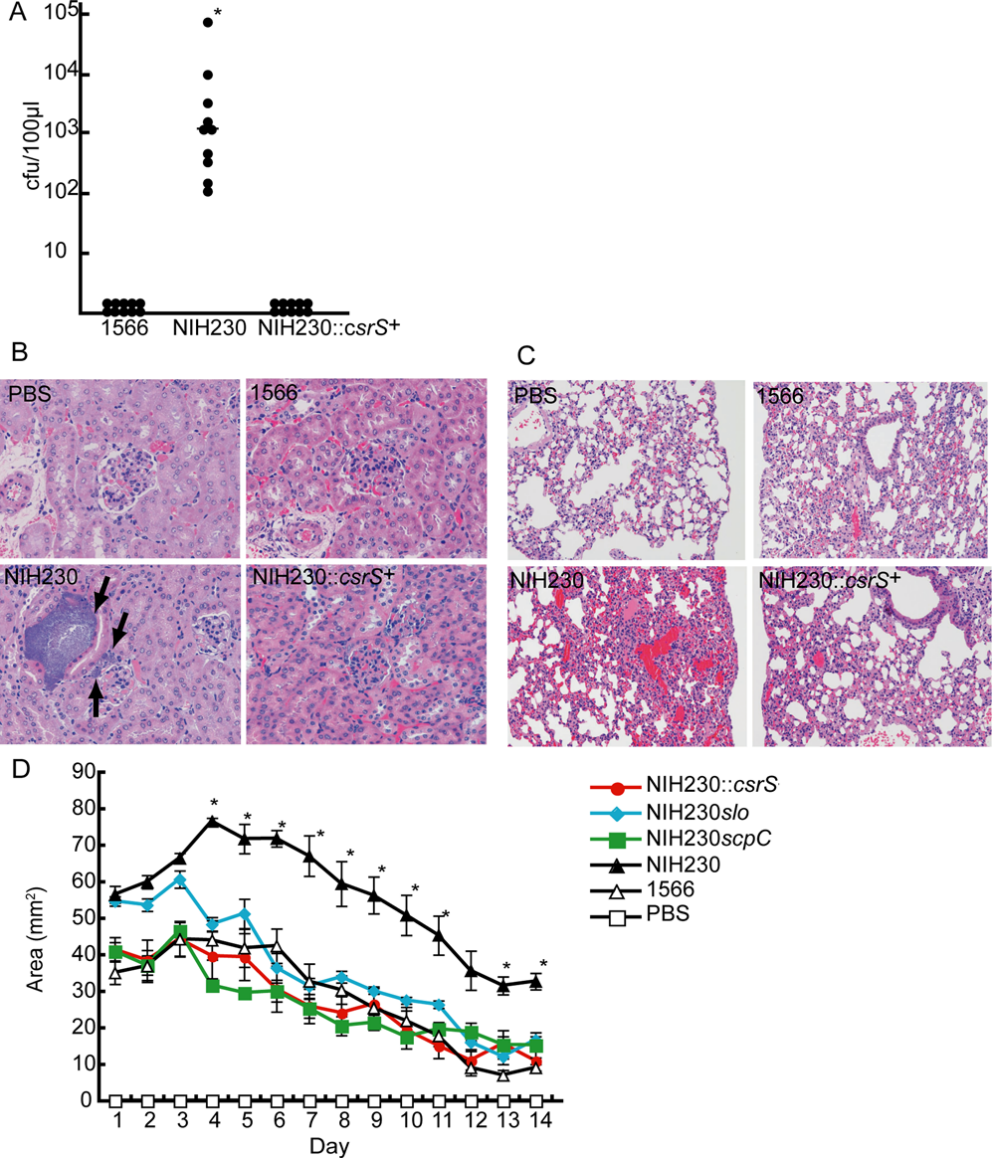
Mutations of the csrS, SLO and ScpC regulate in vivo virulence of GAS in a mouse model. (A) Number of GAS organisms recovered from the blood (100 µL) of each Male ddY mice injected intravenously with 1×10^7^ CFU in 100 ml suspension of GAS in PBS. Blood was taken 24 h after injection and the bacterial count was determined after plating on agar. The (-) bar represents median values. *(p<0.05) estimated by ANOVA. Histopathological changes in the (B) kidney and (C) lungs of mice infected with GAS. Each tissue was extracted 24 h after injecting GAS (1×10^7^ CFU). The black arrows indicate clusters of bacteria. (D) The course of subcutaneous infection in hairless mice injected with 1×10^7^ CFU in 100 µL suspension of GAS in PBS. Lesion area and body weight were measured daily after infection. Values are mean±SEM (n = 5). *Area of skin lesion in NIH230 infected mice was significantly higher than all other groups (p<0.05) estimated by ANOVA.

As shown in Figure 7A, the NIH230 strain caused bacteremia in mice 24 h after intraperitoneal injection whereas the bacteremia was barely detected in mice infected with NIH230::*csrS^+^*as well as the 1566 strain (p = 0.005 compared with non-invasive isolates, and p = 0.005 compared with invasive isolates +CsrS). Histopathologically, in the mice injected with the NIH230 strain, bacteria formed clusters in interstitial tissues in the kidneys and the lungs, accompanied congestion and no inflammatory cells at infectious foci (Figure 7B, 7C and data not shown). Contrarily, no significant pathological alterations were observed in the mice injected with the 1566 and NIH230::*csrS* ^+^ strains (Figure 7B and 7C). Figure 7D shows that subcutaneous infection of NIH230 formed the infected lesions with area significantly larger than those of 1566 and NIH230::*csrS* ^+^. These results suggest that the invasive GAS isolates are more virulent *in vivo* than non-invasive GAS, and impair PMN function *in vivo*, owing, at least in part, to the mutations in the *csrS* gene.

### *scpC* and *slo* are insufficient singly for the pathogenesis of invasive infections

Finally, we assessed the influence of enhanced expression of the *scpC* or the *slo* gene on the virulence in a mouse model. As shown in Table 1, NIH230*scpC* and NIH230*slo* exerted the LD_50_ value 3–10 fold lower than that of the non-invasive isolate 1566, but 10–30 fold higher than that of severe invasive isolates. Subcutaneous inoculation of NIH230*scpC* and NIH230*slo* yielded the local infected lesions with area comparable to those of 1566 and NIH230::*csrS* ^+^ during the course of infection (Figure 7D). These results suggest that enhanced expression of ScpC and SLO in invasive GAS plays an important role *in vivo* virulence of GAS infection.

## Discussion

It have been demonstrated that CsrS/R is a member of the two-component regulatory systems for regulating the multipe virulence factors of GAS, by using genetically- manipulated GAS mutants [20]. The present study demonstrate that the loss-of-functional mutations in *csrS* gene which were accumulated in clinically isolated GAS from patients with severe invasive infections, but not with *emm*-matched non-invasive strains. The *csrS* mutations enhanced the expression of *scpC* and *slo*, associated with the evasion of PMN functions and *in vivo* virulence. Introduction of the intact *csrS* gene into the severe invasive GAS restored the susceptibility to the killing by PMN and abrogated the activity for inhibition of PMN recruitment and survival, thus, demonstrating an instructional role of the loss-of-functional mutations in *csrS* gene for the evasion of PMN functions, providing unique pathophysiology of invasive GAS infections. Previous studies using animal-passaged GAS have shown that mutations in both *csrS* and *csrR* gene are important for the invasive phenotype [5], [6], and mutation frequency of *csrS* and *csrR* seems to be the same [21]. However, the severe invasive isolates analyzed in this study accumulated mutations in the *csrS* gene but not in the *csrR* gene (Figure 5B and data not shown). Then we further examined whether severe invasive isolates of other than emm49 genotype have the mutation of the *csrS* and the *csrR* genes. The frequency of the mutation in the *csrS* gene is higher than that in *csrR* (*csrS* mutation∶*csrR* mutation = 59∶19) (manuscript in preparation), suggesting the *csrS* mutation is more important in comparison with that of *csrR* in the clinical isolates regardless of *emm* genotypes. Furthermore, the expression of some human invasive disease-associated genes [5] including *slo* was enhanced in the *csrS* mutant (Figure 5B), but not in the *csrR* mutant [20]. On the contrary of a dogma that CsrS/R is a definitive member of the two-component regulatory systems, which involve a coordinate pair of proteins known as the sensor kinase and the response regulator [20], CsrS may transmit a signal not only to CsrR but also to other regulators. This dominant role of CsrS is the first important observation in this study, and its mutation is possibly more important than that of *csrR* in terms of etiopathogenesis of human severe invasive diseases.

Numbers of studies has pointed out virulent factors to evade host defense using genetically-manipulated GAS and animal models [18], [22], [23], although the significance of each factor to invasive infection is diverse and sometimes controversial, perhaps due to lack of proper non-invasive counterpart. As examples, SpeB [22] and SLS [23] have been proposed as an invasive infection-associated factor by its cytotoxic effect, however, *speB* and *sagA* expression is not enhanced in any *csrS*-mutated severe invasive GAS isolate used in this study and others [5], [24]. Furthermore, SLS hemolytic activity of invasive GAS is significantly decreased as compared with non-invasive strains (data not shown) and SLS-deletion in invasive GAS did not affect PMN survival at all, excluding the possibility for the role of SLS in PMN necrosis seen in this study. Extracellular deoxyribonuclease (DNase) is a virulence factor that protects *emm1* type GAS against neutrophil killing by degrading the DNA framework of neutrophil extracelluar traps (NETs) [25], [26]. However, we confirmed that addition of DNase in the culture did not alter the level of PI-positive PMN, meaning bright PI staining of PMN is not due to release of NETs from PMN (Figure S1A). DNase activity of the *emm49* severe invasive GAS was lower than that of non-invasive GAS (Figure S1B), possibly due to the difference of *emm* type. The expression of DNase as well as the *slo* and the *scpC* genes in *emm1*-genotype strains was enhanced under the *csrS* mutation [5]. These suggest that DNase may be important but redundant for induction of invasive diseases. Therefore, the second important observation in the present study is that an essential requirement of *csrS* mutation for invasive infection is associated with increased expression of ScpC and SLO and *in vitro* evasion of PMN functions, though we do not exclude the possibility that other CsrS-regulating factors contribute to the escape of invasive GAS from host defense.

SLO and ScpC independently enable GAS to escape from PMN functions; Present data using clinical isolated GAS and a *scpC*-deletion mutant (Figure 4A and 4B) show that enhanced production of serine proteinase ScpC in virulent GAS is essential to impair PMN migration *in vitro* by degradation of IL-8, as others partially have demonstrated [17]–[19]. The present study also uncovers that increased activity of SLO from invasive GAS isolates induces rapid and extensive necrosis to human PMN. SLO is a cholesterol-binding pore-forming hemolysin as well as cytotoxic for other cells [15]. A study has demonstrated SLO from invasive GAS lyse PMN [27], however this effect is likely due to complement activation by SLO [28] or PMN activation [29] but not due to cytotoxity of SLO itself as judged by their flow cytometry profiles which are distinct from ours (Figure 2A). In the present study, we observed that SLO concentration in a short-time culture with severe invasive GAS did not reach the threshold level to kill PMN by formation of pores (data not shown) and that PMN did not undergo necrosis upon incubation with culture media of severe invasive GAS (Figure 3B), leading to the novel possibility that PMN are probably killed following encounter with invasive GAS in a contact-dependent manner. PMN-binding GAS may make a small interface containing a high concentration of SLO between bacteria and PMN, which resembles to killing mechanism of killer cells to target cells [30]. Collaboration of SLO with other toxins may be critical to induce PMN necrosis as similarly mechanism has been reported [31], although it remains to be examined whether there exist explore interaction-associated molecules on both host and bacterial membrane is needed.

In contrast to the previous view [18], we observed that both of ScpC and SLO together, but not each of them, mediated sufficient *in vivo* virulence (Table 1), thus compatible with the notion that plural virulence-associated factors under the regulation of *csrS* abrogate PMN bactericidal functions and induce invasive diseases in *in vivo* animal model. Consistently, the high mortality and histopathological findings which lacks PMN infiltration in mice tissues infected with *csrS*-mutated GAS (Figure 7) are similar to those seen in clinical invasive GAS infections [32]. Thus, these results suggest that the ability of incompetence for PMN functions by individual GAS strain may determine the induction and clinical outcome of invasive diseases. Several clinical reports seem to support this hypothesis; Leukocytopenia seen in patients with STSS is more severe than that with non-STSS [33], and invasive GAS-infected patients with leukocytopenia show worse prognosis than those without leukocytopenia [33], [34]. Furthermore, predisposing factors for severe invasive GAS infection [9], such as diabetes mellitus [35], liver cirrhosis [36], and congestive heart failure [37] are known to impair PMN function. These evidences suggest that the level of PMN function is one of the critical factors to determine the threshold for the onset of invasive GAS infection, which may be the reason for rare outbreaks of invasive GAS infections.

Thus, enhanced expression of virulence factors that could evade PMN function is a key issue at first step to cause invasive bacterial infections. A further study in which collates clinical with bacterial/immunological data may provide with novel clues for early diagnosis and therapeutics of invasive bacterial infections.

## Methods

### Bacterial strains and culture

The *S. pyogenes* strains used in this study are described in Table S1 [8], [38]. *Escherichia coli* DH5α was used as the host for plasmid construction and was grown in liquid Luria-Bertani medium with shaking or on agar plates at 37°C. *S. pyogenes* was cultured in Todd-Hewitt broth supplemented with 0.5% yeast extract (THY medium) without agitation or on tryptic soy agar supplemented with 5% sheep blood. Cultures were grown at 37°C in a 5% CO_2_ atmosphere. When required, antibiotics were added to the medium at the following final concentrations: erythromycin, 500 µg/mL for *E. coli* and 1 µg/mL for *S. pyogenes*; spectinomycin (Sp), 25 µg/mL for *E. coli* and *S. pyogenes* both. The growth of *S. pyogenes* was turbidimetrically monitored at 600 nm using MiniPhoto 518R (Taitec, Tokyo, Japan).

### Animals

Male 5–6-week-old outbred ddY and hairless mice were purchased from SLC (Shizuoka, Japan) and were maintained in a specific pathogen-free (SPF) condition. All animal experiments were performed according to the guidelines of the Ethics Review Committee of Animal Experiments of the National Institute of Infectious Diseases, Japan.

### Isolation of human PMN

PMN were taken from nine healthy volunteers which were composed of 25–52 years old, 7 males and 2 female, and were isolated from venous blood of them using in accordance with a protocol approved by the Institutional Review Board for Human Subjects, National Institute of Infectious Diseases.

### DNA manipulation

DNA amplifications by PCR, DNA restriction-endonuclease digestions, ligations, plasmid preparations, and agarose gel electrophoresis were performed according to standard techniques [42]. PCR reactions were performed using TaKaRa Ex Taq (TaKaRa Bio, Tokyo, Japan). Nucleotide sequence was determined by using the automated sequencer ABI PRISM 3100 Genetic Analyzer (Applied Biosystems, Tokyo, Japan)

### Transformation

Calcium chloride (CaCl_2_) competent *E. coli* cells were prepared and transformed according to a standard protocol [39]. Electrocompetent *S. pyogenes* cells were prepared as described [38].

### Construction of deletion or deficient mutants

1. Construction of the *csrS* mutants. A 1002-bp DNA fragment containing the 5′ terminal of *csrS* and the adjacent upstream chromosomal DNA was amplified from the 1566 chromosomal DNA using the primers for *csrSdel1* and *csrSdel2* (Table S2), and a 1104-bp fragment containing the 3′ terminal of *csrS* and the adjacent downstream chromosomal DNA was amplified from the NIH230 chromosomal DNA using the primers for *csrSdel3* and *csrS4del4* (Table S2); these 2 PCR products were ligated by *Bam*HI and *Eco*RI and by *Eco*RI and *Pst*I, respectively. The digested fragments were cloned into the erythromycin-resistant and temperature-sensitive shuttle vector pJRS233 [40] in order to create the plasmid pJRSΔ*csrS*. This plasmid was then purified from *E. coli* and introduced into the strains NIH230 and 1566 by electroporation. The transformants were selected by observing the growth on erythromycin agar at 30°C. The cells in which pJRSΔ*csrS* had been integrated into the chromosome were selected by the growth of the transformants at 39°C with erythromycin selection. The plasmid-integrated strain was serially passaged in a liquid culture at 30°C without erythromycin selection in order to facilitate the excision of the plasmid and thus, leaving the desired mutation in the chromosome. The replacement of the native *csrS* gene by the *csrS*-deleted mutant allele was verified by PCR, and the resultant strains were named as NIH230Δ*csrS* and 1566Δ*csrS*, respectively.
2. Construction of the *slo* mutant. A 1061-bp DNA fragment containing the internal region of *slo* was amplified from the NIH230 chromosomal DNA using the primers for *slo-del3* and *slo-del4* (Table S2). The PCR products were ligated by *Bam*HI and *Eco*RI. This fragment was then cloned into the integration shuttle vector pSF152 [41] to create the plasmid pSF152*slo* that was then used for the chromosomal inactivation of the *slo* gene, as described previously [40]. The inactivated mutant strain NIH230*slo* (*slo*::*aad9* Sp^r^) was then selected by using spectinomycin-containing agar plates. Deficiency of the native *slo* gene was verified by PCR. Loss of SL hemoltitic activity of these mutants was confirmed by the standard SLS hemolysis assay (Figure 3C).
3. Construction of the *sagA* mutants. A 635-bp DNA fragment containing the 5′ terminal of *sagA* and the adjacent upstream chromosomal DNA was amplified from the NIH230 chromosomal DNA using the primers for sagA0-Xb and sagA2-Bm (Table S2) and a 1037-bp fragment containing the 3′ terminal of sagA and the adjacent downstream chromosomal DNA was amplified from the NIH230 chromosomal DNA using the primers for sagA3-Bm and sagA4-Ps; these 2 PCR products were ligated by *Xba*I and *Bam*HI and by *Bam*HI and *Pst*I, respectively. The digested fragments were then cloned into the temperature-sensitive shuttle vector pJRS233 to create the plasmid pJRSΔsagA that was then used to create NIH230ΔsagA, as described above. Loss of SLS hemoltitic activity of these mutants was confirmed by the standard SLS hemolysis assay.
4. Construction of the *scpC* mutant. A 1240-bp DNA fragment containing the internal region of *scpC* was amplified from the NIH230 chromosomal DNA using the primers for *scpC-del5* and *scpC-del6* (Table S2). The PCR products were ligated by *Bam*HI and *Eco*RI. This fragment was then cloned into the integration shuttle vector pSF152 [41] to create the plasmid pSF152*scpC* that was then used to create NIH230*scpC*, as described above.

### Construction of the strains integrating the intact *csrS* gene

The *csrS* gene replacement was performed by allelic recombination. Specifically, the chromosomal DNA derived from the GAS strain 1566 was purified and used as a template for the PCR amplification of the *csrS* gene. The primers used were 5′-GGGGATCCTGAGATTCCTCTCACTAAAC-3′ (sense) and 5′-GGGAATTCTCTAATACACTATTTTACC-3′ (antisense). The PCR fragment was ligated into the plasmid pSF152 [41], and the resultant plasmid pSF*csrS* was used for chromosomal integration into the mutated *csrS* gene of isolates from patients of severe invasive infections, as described previously [41]. The integrated strains (Sp^r^) were then selected by using spectinomycin (Sp)-containing agar plates. The integration of the *csrS* gene was confirmed by PCR.

### RT-PCR

*S. pyogenes* was grown in THY media at 37°C without aeration, and total RNA was extracted at OD_600_ of 0.75 by using the RNeasy Mini extraction kit (Qiagen). RT-PCR was performed by using a One Step RNA PCR Kit (AMV) (TaKaRa Shuzo Co., Kyoto, Japan) according to the manufacturer's recommendation using the RT-PCR primer pairs shown in Table S3.

### GAS infection in a mouse model

To determine LD_50_, We injected several dilutions of 0.5 mL of GAS isolate suspensions in phosphate-buffered saline (PBS) intraperitoneally into male 5–6-week-old ddY outbred mice (5 mice/dilution, 9 dilutions for each GAS isolate). The exact numbers of the colony-forming units of the injected bacteria were determined by incubating adequate dilutions of each GAS sample on sheep blood agar plates. The data were analyzed for significance according to the Probit method to determine the LD_50_ values for a 7-day period. For a subcutaneous infection model, male hairless mice Hos:Hr-1 were injected 1×10^7^ CFU in 100 µl suspension of GAS in PBS. Lesion area and body weight were measured daily, and analyzed

### Histopathology

For histological analysis, the tissues from GAS-infected mice were fixed in 10% formalin/PBS. The paraffin-embedded sections were stained with hematoxylin and eosin (Sapporo General Pathology Laboratory Co. Ltd., Hokkaido, Japan).

### Phagocytosis and killing assay

Phagocytosis and killing assay by PMN were performed as previously described with some modifications [14]. 5×10^5^ human PMN and 5×10^6^ bacteria opsonized with human plasma and labeled with alexa 488 (Invitrogen, Carlsbad, CA) in a well of 24 well plates. After incubation for 60 min, PMN were harvested and stained with alexa-594 labeled anti-alexa 488 polyclonal Ab (Invitrogen) in order to distinguish non-phagocytosed but attached bacteria to PMN. The proportion of phagocytosed PMN were analyzed by FACS Calibur (BD Biosciences, San Jose, CA), For killing assay, PMN and opsonized bacteria in the same MOI as phagocytosis assay were incubated for 2 hours at 37°C, adding antibiotics at 60 min to eliminate non-phagocytosed bacteria. Corrected PMN were lysed in 0.1% saponin / PBS for 20 min on ice. Bacteria were washed with PBS and cultured on soy beans agar plate overnight, for counting the number of colonies.

### Migration assay

Chemotaxis assay were performed as previously described with modification [42]. Briefly, 5×10^5^ PMN in RPMI medium containing 25 mM HEPES and 1% FCS were in Transwell inserts (3 µm pore size, Coaster, Corning, NY) placed in 24-well plates containing 600 µl medium, or 100 nM IL-8 solution (Peprtec, London, UK), which were incubated with or without 5×10^6^ bacteria for 1 hour at 37°C in advance of the assay. After 1 hour incubation, cells in the lower wells were collected and 10^4^ 10 µm microsphere beads (Polysciences Inc., Warrington, MA) were added. Cells were stained with propidium iodine (Sigma, St Louis, MI) for flow cytometry to quantify viable PMN and were analyzed using FACSCalibur. In some experiments, cholesterol (Sigma), 25 µg/mL anti-SLO polyclonal Ab (American Research Product, Inc., Belmont, MA), or rabbit IgG was added in 24 well plates.

### ELISA

The amount of IL-8 in supernatant after incubation with bacteria was determined by Ready-to-Go human IL-8 ELISA kit (eBioScience, San, Diego, CA) according to manufacturers' protocol.

### SLO-hemolysis assay

The activity of SLO in supernatant is measured as previously described [43]. Briefly, overnight culture supernatants of various strains were subjected to centrifugation, and were filtrated through a 0.45 µm membrane. Dithiothreitol and trypan blue were then added to each sample to a final concentration of 4 mM and 13 µg/mL respectively, and the mixtures were incubated at room temperature for 10 min. A 0.2 ml aliquot of 5% (v/v) sheep erythrocyte in PBS was added to 0.4 ml of each treated sample. After 30 min incubation at 37°C, the mixtures were subjected to centrifugation, and absorbance of the supernatants fluids was measured at 540 nm. To confirm that hemolysis was due to SLO, control reaction including culture supernatants to which water-soluble cholesterol (Sigma), a specific SLO inhibitor, had been added to yield a final concentration of 250 µg/mL.

### SLS-hemolytic assay

Overnight culture of various strains were frozen at −80°C, thawed, and centrifuged to obtain the supernatants. Serially diluted culture supernatants (0.1 ml) in PBS containing 250 µg/mL water-soluble cholesterol were incubated at room temperature for 10 min. A 0.1 ml aliquot of 5% (v/v) sheep erythrocyte was added and incubated for 1 h at 37°C. The mixture were subjected to brief centrifugation, and absorbance of the supernatants fluids was measured at 540 nm. To confirm that hemolysis was due to SLS, control reaction including culture supernatants to which trypan blue (Sigma), a specific SLS inhibitor, had been added to yield a final concentration of 13 µg/ml.

## Supporting Information

Figure S1 DNase activity is not involved in the virulence of emm49 severe invasive GAS isolates. a) To investigate the role of DNase in PMN survival, the viability of PMN that migrated in lower wells of transwell system was estimated as PMNs were applied into the upper well (5×105 cells) of a transwell system, and lower wells consisted of IL-8 in the presence or absence of DNase I (100 mg/ml at a final concentration), together with either non-invasive GAS (1566), or invasive GAS (NIH230). PMN migrated in lower wells were stained with propidium iodine and were analyzed using flow cytometry. b) Activity of DNase in emm49 GAS. 10 ng of Calf thymus DNA was incubated with or without culture supernatants from non-invasive, severe invasive, and CsrS-transduced severe invasive GAS for 15 min at 37°C. Activity to degrade calf thymus DNA was visualized by 1% agarose gel electrophoresis. Methods in vitro migration assay As shown 5×105 PMN in RPMI medium containing 25 mM HEPES and 1% FCS were in Transwell inserts (3 µm pore size, Coaster) placed in 24-well plates containing 600 µl medium, 100 nM IL-8 solution (Peprtec), 100 µg/mL deoxyribonuclease I (Sigma, St Louis, MI) which were incubated with or without 5×106 bacteria for 1 hour at 37°C in advance of the assay. After 1 hour incubation, cells in the lower wells were collected and 104 10 µm microsphere beads (Polysciences) were added. Cells were stained with propidium iodine (Sigma) for flow cytometry to quantify viable PMN and were analyzed using FACSCalibur (BD BioScience). DNase activity assays Supernatants were collected from overnight cultures of bacterial strains grown in THB. Calf thymus DNA (10 ng) was combined with bacterial supernatant in final volume of 50 ml buffer (300 mM Tris-HCl (pH 7.5), 3 mM CaCl2, 3 mM MgCl2) for 15 min at 37°C. To halt DNase activity, 10 ml of 0.5 M EDTA (pH 8.0) was added to the reaction. Visualization of DNA degrad tion was done in 1% agarose gel electrophoresis.
(0.60 MB TIF)

Table S1 Strains and plasmids used in this study
(0.03 MB DOC)

Table S2 Primers used for the construction of deletion mutants
(0.03 MB DOC)

Table S3 Primers used in RT-PCR
(0.03 MB DOC)
