# Supplementary figures and images for: Incompetence of Neutrophils to Invasive Group A streptococcus Is Attributed to Induction of Plural Virulence Factors by Dysfunction of a Regulator
Source: PLoS One. 2008 Oct 21;3(10):e3455. doi: 10.1371/journal.pone.0003455 (PMC2565068; doi:10.1371/journal.pone.0003455)

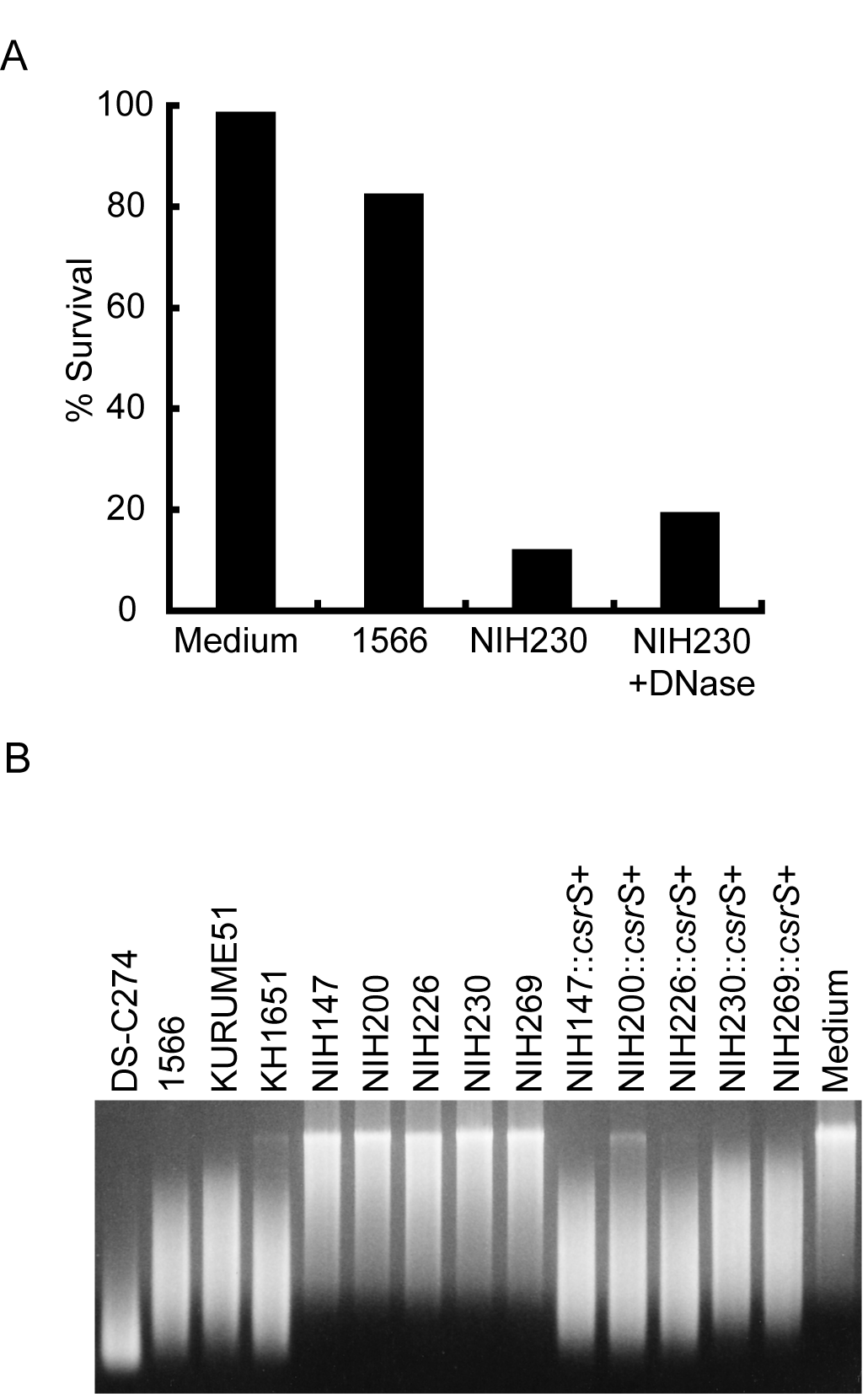

Supplement: Figure S1 — DNase activity is not involved in the virulence of emm49 severe invasive GAS isolates. a) To investigate the role of DNase in PMN survival, the viability of PMN that migrated in lower wells of transwell system was estimated as PMNs were applied into the upper well (5×10^5^ cells) of a transwell system, and lower wells consisted of IL-8 in the presence or absence of DNase I (100 mg/ml at a final concentration), together with either non-invasive GAS (1566), or invasive GAS (NIH230). PMN migrated in lower wells were stained with propidium iodine and were analyzed using flow cytometry. b) Activity of DNase in emm49 GAS. 10 ng of Calf thymus DNA was incubated with or without culture supernatants from non-invasive, severe invasive, and CsrS-transduced severe invasive GAS for 15 min at 37°C. Activity to degrade calf thymus DNA was visualized by 1% agarose gel electrophoresis. Methods in vitro migration assay As shown 5×10^5^ PMN in RPMI medium containing 25 mM HEPES and 1% FCS were in Transwell inserts (3 µm pore size, Coaster) placed in 24-well plates containing 600 µl medium, 100 nM IL-8 solution (Peprtec), 100 µg/mL deoxyribonuclease I (Sigma, St Louis, MI) which were incubated with or without 5×10^6^ bacteria for 1 hour at 37°C in advance of the assay. After 1 hour incubation, cells in the lower wells were collected and 104 10 µm microsphere beads (Polysciences) were added. Cells were stained with propidium iodine (Sigma) for flow cytometry to quantify viable PMN and were analyzed using FACSCalibur (BD BioScience). DNase activity assays Supernatants were collected from overnight cultures of bacterial strains grown in THB. Calf thymus DNA (10 ng) was combined with bacterial supernatant in final volume of 50 ml buffer (300 mM Tris-HCl (pH 7.5), 3 mM CaCl2, 3 mM MgCl2) for 15 min at 37°C. To halt DNase activity, 10 ml of 0.5 M EDTA (pH 8.0) was added to the reaction. Visualization of DNA degrad tion was done in 1% agarose gel electrophoresis. (0.60 MB TIF) [file pone.0003455.s001.tif]
