# Supplementary material for: Incompetence of Neutrophils to Invasive Group A streptococcus Is Attributed to Induction of Plural Virulence Factors by Dysfunction of a Regulator
Source: PLoS One. 2008 Oct 21;3(10):e3455. doi: 10.1371/journal.pone.0003455 (PMC2565068; doi:10.1371/journal.pone.0003455)
Supplement: Table S1 — Strains and plasmids used in this study (0.03 MB DOC) [file pone.0003455.s002.doc]

Table S1 Strains and plasmids used in this study

| Strain name | Relevant characteristics^a^ | Reference |
| --- | --- | --- |
| DS-C274 | nephritis | 41 |
| 1566 | pus | 41 |
| KURUME51 | nephritis | 41 |
| K.H1651 | nephritis | 41 |
| NIH147 | NF, ARI | 41 |
| NIH200 | STSS | 41 |
| NIH226 | NF, ARI, LC | 41 |
| NIH230 | STSS | 41 |
| NIH269 | STSS | 41 |
| NIH147::*csrS^+^* | NIH147 carrying intact *csrS*^+^, Sp^r^ | This study |
| NIH200::*csrS^+^* | NIH200 carrying intact *csrS*^+^, Sp^r^ | This study |
| NIH226::*csrS^+^* | NIH226 carrying intact *csrS*^+^, Sp^r^ | This study |
| NIH230::*csrS^+^* | NIH230 carrying intact *csrS*^+^, Sp^r^ | This study |
| NIH269::*csrS^+^* | NIH269 carrying intact *csrS*^+^, Sp^r^ | This study |
| 1566△*csrS* | 1566 carrying *csrS* deletion mutation | This study |
| NIH230△*csrS* | NIH230 carrying *csrS* deletion mutation | This study |
| NIH230*slo* | NIH230 carrying *slo* deficient mutation | This study |
| NIH230*scpC* | NIH230 carrying *scpC* deficient mutation | This study |
| NIH230*slo sagA* | NIH230 carrying *slo* and *sagA* deficient mutation | This study |
| NIH230*sagA* | NIH230 carrying *sagA* deletion mutation | This study |
| plasmid name | Relevant characteristics |  |
| pJRS233 | temperature-sensitive shuttle vector | 43 |
| **pSF152** | integration shuttle vectors | 44 |

a: Strains were isolated from symptomatic patients.

ARI, acute renal insufficiency; LC, liver cirrhosis; NF, necrotizing fasciitis; STSS, streptococcal toxic shock-like syndrome^47^
