# Supplementary material for: Incompetence of Neutrophils to Invasive Group A streptococcus Is Attributed to Induction of Plural Virulence Factors by Dysfunction of a Regulator
Source: PLoS One. 2008 Oct 21;3(10):e3455. doi: 10.1371/journal.pone.0003455 (PMC2565068; doi:10.1371/journal.pone.0003455)
Supplement: Table S2 — Primers used for the construction of deletion mutants (0.03 MB DOC) [file pone.0003455.s003.doc]

Table S2 Primers used for the construction of deletion mutants

| Primer | Sequence (5′–3′) |
| --- | --- |
| csrSdel1 | GGGGATCCTAATCCTTTTGCTAGCTTG |
| csrSdel2 | GGGAATTCCATATGACTTATTTCTCAC |
| csrSdel3 | GGGAATTCATAGAGTAGCTTAAGGCATTG |
| csrSdel4 | GGCTGCAGTATTTCTTGCTTGACTTGGG |
| sagA0-Xb | GGGGTCTAGAGGATGTTCCACCAGTGGACT |
| sagA2-Bm | GGGGATCCCATAAGGTTTACCTCCTTATC |
| sagA3-Bm | GGGGATCCTAATCTATTTAGCATCTCTATG |
| sagA4-Ps | GGCTGCAGTCAATTGAATATTCTGGG |
| slo-del3 | GGGGATCCCTCCCAAAGAAATGCCAC |
| slo-del4 | GGGAATTCTGAAATAGGATAAGCTGGG |
| scpC-del5 | GGGGATCCGTGGCTTTAGGAGCAGATGTG |
| scpC-del6 | GGGAATTCGAGAAGTCAAGGTGAAGCGG |
