# Supplementary material for: Incompetence of Neutrophils to Invasive Group A streptococcus Is Attributed to Induction of Plural Virulence Factors by Dysfunction of a Regulator
Source: PLoS One. 2008 Oct 21;3(10):e3455. doi: 10.1371/journal.pone.0003455 (PMC2565068; doi:10.1371/journal.pone.0003455)
Supplement: Table S3 — Primers used in RT-PCR (0.03 MB DOC) [file pone.0003455.s004.doc]

Table S3 Primers used in RT-PCR

| Gene | Primer name | Sequence (5′–3′) |
| --- | --- | --- |
| *mac* | ideS-F | GGTAGATCGTGGTGTTATAG |
|  | ideS-R | TTAGCGTAGGTGTGTGATAG |
| *nga* | NAD5′ | ATGAGAAACAAAAAAGTAAC |
|  | NAD3′no.1 | TTACTTCCTATCTTGC |
| *slo* | slo-1 | CTTATCCTATTTCATACACC |
|  | slo-2 | CTACTTATAAGTAATCGAACC |
| *scpC* | prtS-F | AAAGGAGCTTGGGACAAGGG |
|  | prtS-R | TGATCCCATGATGTCGTTGG |
| *hasA* | hasA-F | TATATTGTTGATGATGGGAG |
|  | hasA-R | AACTTTTTAATTGGAAAGG |
| *scpA* | scpA1 | CCATTTGATAAACTTGCC |
|  | scpA2 | ATTAATCACCTTAGCTCCC |
| *speB* | SPE-B1 | GATCAAAACTTTGCTCGTAACG |
|  | SPE-B2 | AGGTTTGATGCCTACAACAGC |
| 16SrRNA | 16S-F | GGGAGGCAGCAGTAGGG |
|  | 16S-R | TAGAGCGGGCATCGGGATG |
